# Editorial: Updates and new concepts in regulation of pro-inflammatory gene expression by steroid hormones, volume II

**DOI:** 10.3389/fendo.2023.1337386

**Published:** 2023-11-30

**Authors:** Isaias Glezer, Cristoforo Scavone, Maria Christina W. Avellar

**Affiliations:** ^1^ Department of Biochemistry, Escola Paulista de Medicina, Universidade Federal de São Paulo, São Paulo, Brazil; ^2^ Department of Pharmacology, Instituto de Ciências Biomédicas, Universidade de São Paulo, São Paulo, Brazil; ^3^ Department of Pharmacology, Escola Paulista de Medicina, Universidade Federal de São Paulo, São Paulo, Brazil

**Keywords:** asthma, cancer, gene expression, glucocorticoid, olfactory, mineralocorticoid, nuclear receptor, phase-separation

In 2018 we concluded the edition of a Research Topic dedicated to articles reporting novel, controversial, or trending information related to the effects of steroid hormone signaling on pro-inflammatory gene expression (1). Two years later, a potentially lethal and highly contagious disease, which in its severe form is essentially an inflammatory syndrome (2), impacted the scientific community and promoted a quest for new knowledge and treatments related on the subject. An unprecedented worldwide acute use of the steroidal anti-inflammatory drug dexamethasone (DEX) during COVID-19 pandemic saved the lives of a significant fraction of patients diagnosed with severe illness (3). This and other recent developments in the field motivated the release of a second volume of the Frontiers Research Topic.

DEX is a glucocorticoid (GC) receptor (GR) agonist that depicts the prototypical anti-inflammatory effect of corticosteroids, but it is also well-known that the endogenous hormone cortisol engages mineralocorticoid receptors (MRs) as well. In this second volume of the Research Topic, Edwards et al. hypothesize that loss of ACE2 activity due to SARS-CoV-2 cell infection promotes ATP release, which leads to excessive reactive oxygen species (ROS) production and oxidative stress. Accordingly, such a condition leads to loss of the normal MR protective mechanism, resulting in receptor activation by cortisol, increased ATP release and respiratory distress. The authors compared patients treated with a high dose of DEX against a low dose of DEX in combination with the MR antagonist spironolactone, and found that the combined treatment was equally effective in treating SARS-CoV2-positive hospitalized patients with pneumonia. In addition, the combined treatment showed some improvement in symptoms and clinical laboratory findings (e.g. D-dimer levels). Lastly, spironolactone/DEX treatment promoted no adverse effects on SARS-CoV-2 outpatients. This article suggests that a parallel pathway activated by cortisol and MR may contribute to the severity of COVID-19, which awaits evaluation of pro-inflammatory gene expression.

Another airway inflammatory disease treated with glucocorticoids is asthma, and treatment resistance is common in patients with severe asthma and in asthmatics who smoke (4). In order to discuss and gain insight into the mechanisms of asthma pathogenesis, Grunstein elaborated a perspective paper on homeostatic glucocorticoid signaling in airway smooth muscle. In this perspective, a pro-asthmatic scenario characterized by pro-inflammatory cytokines and β2-adrenergic receptor desensitization, activates mitogen-activated protein kinase (MAPK) signaling and contributes to local GC-unbound GR activation and conversion of endogenous cortisone to cortisol, leading to a feedback inhibition via MAPK phosphatase. The author discusses the rationale behind prescribed GC therapy and the consequences of disrupting this homeostatic mechanism in asthma, pointing out the delicate balance of GC hormone signaling in health and disease.

Interestingly, GC signaling changes during cancer development and progression. While GCs may restrain inflammatory damage and provide protection against neoplasia development, such protective functions are reduced or may be reversed during chronic inflammation. The pro- and anti-tumor effects of GC signaling can be hard to interpret, especially taking into account how GR agonists are prescribed to patients with solid cancers. In fact, GC signaling in solid tumors may be obscured by the fact that exogenous GCs are commonly co-administered with anticancer therapies to alleviate unwanted side effects. Khadka et al. discuss this complex therapeutic reality, and reviewed the endocrine aspects linked to HPA-axis on immune cells associated with tumors, including how exogenous GCs and the interplay with other steroid hormone receptors, interfere with GR-triggered downstream effects on neoplasia progression. We may expect that future developments in this field tackle these relevant ambiguities, providing new mechanisms for improved therapies and drug design.

Most reviews on GR signaling cover the basic dynamics involved of GR activation, its effects on chromatin remodeling, binding to regulatory elements and other transcription factors, and recruitment of the transcriptional machinery. Less explored is the biophysical interplay between GR and other DNA-associated macromolecules that promotes liquid-liquid(phase-separation (LLPS), and how this phenomenon impacts gene expression (Pinheiro et al.). Since this is an emerging topic in biology and few studies have been conducted specifically with GR, Pinheiro et al. reviewed what is known about GR and other steroid hormone nuclear receptors LLPS on chromatin topology and gene expression. The article describes how different studies point towards a role for intrinsically disordered regions as drivers of GR condensation with other proteins at DNA level. LLPS can impact the different modalities of GR action on gene expression, for instance, through transcription active or transcript inactive condensates, which are plausible molecular events in glucocorticoid signal transduction. Future studies are necessary to determine if this mechanism will impact our knowledge on endocrine control of inflammation, which can impact inflammatory syndromes and diseases, as well as new strategies for cancer control and prevention.

## Author contributions

IG: Writing – original draft, Writing – review & editing. CS: Writing – review & editing. MA: Writing – review & editing.
